# Pyrolysis, a recovery solution to reduce landfilling of residual organic waste generated from mixed municipal waste

**DOI:** 10.1007/s11356-024-33282-1

**Published:** 2024-04-13

**Authors:** Jessica Graça, Marzena Kwapinska, Brian Murphy, Tim Duggan, James J. Leahy, Brian Kelleher

**Affiliations:** 1 School of Chemical Sciences, Dublin City University, Dublin 9, Ireland; 2 Department of Chemical Sciences, University of Limerick, Limerick, V94 T9PX Ireland; 3 Enrich Environmental Ltd, Larch Hill, Kilcock, Co Meath Ireland; 4 Department of Chemical Sciences, Bernal Institute, University of Limerick, Limerick, V94 T9PX Ireland

**Keywords:** OFMSW, BSRW, Residual organic waste, Biochar, Pyrolysis gas, Circular Economy

## Abstract

Despite policies to restrict the mixing of organic waste with other general waste and improve its separation at source, municipal solid waste still contains a high proportion of organic waste. The residual organic waste is generated as a by-product of the mechanical treatment of municipal solid waste (MSW) and is mainly disposed in landfills after composting. Its reuse and recovery status varies across European countries. Most countries restrict the use of biostabilised residual waste (BSRW) to landfill cover, whereas others have regulated it as marketable compost. Crucially, BSRW is set to lose its “recycled” status under the revised European Union waste framework, with probably tighter restrictions and increased costs imposed for the landfilling of organic waste. Our research aimed to investigate pyrolysis as an alternative technology to treat the 10–40 mm fraction of BSRW (representing 50% of BSRW generated). Pyrolysis at 700 °C was carried out and feedstock and pyrolysis products were characterized. Mass and energy balances showed that pyrolysis produced hot vapour/gas whose combustion may render the pyrolysis process energetically sustainable. Biochar comprises 30–50% of BRSW mass after removal of glass, metal and stones. Our results indicate that pyrolysis has the potential to create options for contributing to reduce the landfilling of BSRW; however, the presence of residual impurities may limit biochar applications.

## Introduction

Residual organic waste is the organic waste that is placed in the general collection and mixed with other wastes. Globally, residual organic waste comprises 44% of the municipal solid waste (MSW), and although developed countries tend to present lower levels of organic waste in the MSW, this fraction is the biggest fraction of MSW throughout developed and undeveloped countries (Kaza et al. 2018). Similarly, in Ireland, ${~}^{2}\!\left/ \!{~}_{3}\right.$  of the composition of general household waste is made up of organic waste, with only 12% being source separated, according to figures for the Irish Environment Protection Agency (EPA) in 2021. The European Union (EU) regulatory framework, the Landfill Directive (Council Directive 1999/31/EC) and the Waste Framework Directive (Directive 2008/104/EC) have imposed restrictions on the landfill of biodegradable waste. These restrict the treatment of the residual municipal solid waste (MSW) in the EU to incineration or landfilling. The residual MSW is deemed unsuitable for landfill disposal without undergoing biological treatment, due to its potential to release odours, greenhouse gases and leachates, incurring greater economic and environmental costs. The residual MSW is therefore subject to a range of mechanical and biological processes prior to final disposal (Graça et al. 2023). In the first screening, the fraction > 80 mm, obtained through mechanical trommel separation, is recovered by co-incineration. The < 80 mm fraction, mechanically separated organic fraction of municipal solid waste (OFMSW), is used as landfill daily cover after going through a composting process. Biostabilised residual waste (BSRW) or municipal compost (Rigby et al. 2021) is referred to as OFMSW after being subject to compost treatment. Pyrolysis is an attractive alternative to incineration that allows the reduction of solid organic waste from 80 to 95% by volume (Basu 2010) and MSW from 50 to 80% by mass (Li and Skelly 2023). The pyrolysis process in the management of residual organic waste has two major potential benefits, the recovery of BSRW reducing its landfilling and its volume and transforming a hard to treat waste into added resources such as biochar and syngas. There are studies that demonstrated the successful production of biochar from fermentation residue (Maroušek 2014) and chicken manures (Morgano et al. 2018), while the pyrolysis gas may be directly combusted to make the process energetically sustainable, and biochar recovered for soil applications.

Biochar can be applied in water and/or slurry treatment, as an additive in animal feedstock, and spread on land to improve carbon stocks (Bartoli et al. 2020; Gwenzi et al. 2021; Jindo et al. 2020a).

Biochar produced from different feedstocks varies widely in properties. For agricultural applications, biochar produced from source-segregated waste such as biomass, crop residues, food processing waste or manures is preferred (Samoraj et al. 2022). There is an unexplored potential for biochar produced from mixed waste, such as BSRW, in environmental applications such as recovery of land contaminated with metals or organic pollutants (Lu et al. 2020b; Wang et al. 2018).

Prior studies showed that BSRW is a heterogeneous material, characterized by a high ash and moisture contents, making it unfavourable for large-scale pyrolysis (Agar et al. 2018; Eke et al. 2020). The authors approach consisted of generating a pyrolysis feedstock from BSRW technically suitable for the process, by selecting the 10–40 mm fraction, which represents at least 50% of BSRW currently generated. The remaining small material (< 10 mm) would be used as a soil amendment (Graça et al. 2023). We hypothesized that due to its energy properties and lower ash and moisture content, the 10–40 mm fraction of BSRW is better suited for energy production and material recovery, which would eliminate its landfilling. The aim of this study was to evaluate the suitability of the BSRW (10–40 mm fraction) as a feedstock for pyrolysis. To the best of the authors’ knowledge, there are no previous studies available on pyrolysis of this BSRW fraction and a blend of material from green waste composting process, Compost Oversize and BSRW. The specific objectives of this study are (1) characterization of BSRW and Compost Oversize properties, (2) characterization of pyrolysis products biochar and syngas and (3) evaluating the mass and energy balance of pyrolysis process.

## Materials and methods

### Pyrolysis feedstock

The main feedstock for pyrolysis was BSRW due to its currently limited reuse and high generation rate. The material was collected after the *in-vessel* composting plant site in Co. Meath, Ireland (Graça et al. 2021). The waste material was derived from the mechanical treatment of residual MSW. Sampling was performed over two seasons, three samples were collected in the summer (May–June) and three over the winter (October–December), to evaluate the potential variability of the feedstock for pyrolysis process (Table 1).

**Table 1:** Overview of the samples subject to analyses and pyrolysis experiment

	Analysis conducted		Pyrolysis
	Impurities	Proximate analysis	
BSRW 070519	✓	✓	
BSRW 040619	✓	✓	
BSRW 210619	✓	✓	
BSRW 291019		✓	✓
BSRW 251119	✓	✓	✓
BSRW 021219		✓	✓
Compost Oversize		✓	✓
1:1 (w/w) BSRW/Compost Oversize			✓

Compost Oversize (> 20 mm) from windrow compost (Compost Oversize) of source-segregated green waste was collected after the composting and refinement of marketable compost. This material is readily available in composting plants, and it was used to blend with BSRW in a 1:1 (v/v) ratio of BSRW and Compost Oversize to study the possibility of generating of biochar with intent to be used as soil amendment.

### Sample collection and preparation

A total of six primary samples of BSRW were obtained after the composting process as outlined in (Campbell et al. 2011). From each BSRW batch, the primary sample was produced as a composite sample of 30 kg, generated by randomly sampling 15 grabs of 2 kg. Each sample was then mixed and sieved at 10 mm. The BSRW 10–40 mm fraction was used for the feedstock analysis (Table 1). Using the quartering and coning technique, 5 kg of the BSRW 10–40 mm fraction was used immediately for determination of moisture (EN 15414–3:2011) and bulk density. Ten litres of each sample was dried at 105 °C overnight and storage for impurities characterization (AfOR 2012, CEN/TS 16202:2013) and a 5 kg of sample was immediately frozen for proximate and characterization analysis and for the pyrolysis study.

### Feedstock impurities and particle size distribution

Particle size distribution and impurities of four BSRW samples were measured simultaneously (Table 1), using AfOR MT PC&S:2002 and CEN/TS 16202:2013 standard procedures. The samples were analyzed in triplicate, using a series of sieves with decreasing apertures (31.5 mm, 16.0 mm, 10.0 mm, 8.0 mm, 5.0 mm, 4.0 mm and 2.0 mm). Impurities classes considered for this study are presented in Table 2. These were separated by hand during the dry-sieving process, weighed and expressed as a percentage of total mass (w/w%). The amount of sample retained was also recorded. In the present study, only data corresponding to the 10–40 mm fraction is considered.

**Table 2:** Impurities classes considered in this study

Impurities class	Description
Inert	Glass, metal, stones, C&D
Glass	Glass only
Metal	Metal, aluminium foil
Stones/C&D	Stones; construction and demolition material
Plastic	Hard and soft plastic, styrofoam, rubber, latex
Paper	Absorbent paper, wipes, printing paper
Others	Textile, bone, electric waste, unusual items such as credit cards

### Pyrolysis procedure

The three winter BSRW 10–40 mm samples were chosen for the pyrolysis experiments, after the moisture content and proximate analysis were performed. The samples collected during the winter season represent less favoured properties for the pyrolysis process, such as slightly lower heating value and higher moisture and ash content. This way, due to the seasonal variability often seen during composting of OFMSW, is it assumed by the authors that the BSRW 10–40 mm samples collected in the summer would be at least equally valuable for pyrolysis.

In addition, the Compost Oversize and the 1:1 BSRW 10–40 mm and Compost Oversized (v/v) were also pyrolyzed. The samples were homogenized, and the particle size was reduced. Glass, metal and construction and demolition material were removed from each sample after defrosting. Immediately prior to the pyrolysis experiment, the moisture and ash content of the samples were measured.

A laboratory-scale fixed bed reactor was used to carry out the pyrolysis experiments as schematically outlined in prior studies (Agar et al. 2018; Simbolon et al. 2023). The main part of the experimental setup was the tubular (45/50 mm, ID/OD) reactor made from quartz (H. Baumbach & Co. Ltd., UK) wrapped with a heating tape (Omegalux, USA) and a high-temperature insulation. The reactor was connected with a condenser cooler and a twin-neck receiving flask (borosilicate glass, Pyrex®, Quickfit®) that enable syngas sampling and collection of pyrolysis liquid. When syngas was not sampled, the outlet of the receiving flask was connected to a filter for capturing aerosols. Temperature inside the reactor was measured using a sheathed K-type thermocouple inserted into the reactor via a feed-through hole in the rubber stopper. Once the temperature inside the reactor reached 700 °C, either 30 g (251119 and 021219 BSWR) or 40 g (291019 BSWR, Compost Oversized, 1:1 BSWR/Compost Oversized) of feedstock in a steal-mesh basket was placed in the reactor. After 10 min the heating was turned off, but the biochar was kept in the reactor until it cooled to room temperature. No nitrogen flow was applied for pyrolysis experiments. However, some air entered the reactor when samples were inserted. Consequently, 10–13 vol. % of nitrogen and about 2 vol. % of oxygen were present in syngas. The syngas after passing through the cooler was collected in a 10 L Tedlar® bag with a polypropylene valve (Restek, Ireland). The biochar yield was calculated as the ratio between the mass of the biochar and the initial mass of the sample in the steal-mesh basket. The pyrolysis experiment for each material was repeated 8 times, and the product yields are the average from all tests. Syngas was sampled at least 2 times, and the average gas composition is reported. Most of the liquid product from pyrolysis was collected in the receiving flask; however, some of the oil/tar condensed on the cooler ends of the experimental setup. The mass of condensed oil/tar was determined by taking the difference between the mass of the reactor and all the connected glassware before and after a series of pyrolysis runs for each material and then added to the mass of the liquid. The mass fraction of syngas was determined as the difference between the initial mass of the feedstock, mass of biochar and mass of liquid. The yield of pyrolysis product was calculated as the mass ratio between the product and the initial mass of feedstock used in the pyrolysis experiments multiplied by 100%.

### Characterization of the feedstock, pyrolysis gas and biochar

Samples of biochar and pyrolysis feedstock were prepared for analysis according to EN 15443:2011. Proximate analysis was carried out according to standard methods for heating value (EN 15400:2011), moisture content (EN 15414–3:2011), volatile matter (EN 15402:2011) and ash content (EN15403:2011). The elemental composition (carbon, nitrogen, hydrogen) of the feedstock material and pyrolysis biochar was determined according to the standard method EN15407:2011, along with sulphur (EN15408:2011) and chlorine content (CA36-WaveDispersive XRF). Proximate and ultimate analyses were carried out by a certified laboratory (SOCOTEC UK Ltd.). Pyrolysis biochar pH and electrical conductivity were determined using a 1:40 sample-to-water ratio (Apaydın-Varol and Pütün, 2012), in duplicate. Major elements (EN 15410:2011) and trace metals (EN 15410:2011) in biochar samples were determined using Inductively coupled Plasma by a certified laboratory (SOCOTEC UK Ltd.). Biochar surface area was determined using a BET surface area analyser (Micromeritics Gemini VII). Syngas composition (carbon dioxide, carbon monoxide, ethylene, acetylene, methane, hydrogen sulphide, hydrogen, nitrogen and oxygen) was analyzed using a micro gas chromatograph (Agilent 3000).

## Results and discussion

### Feedstock properties

Currently, the reuse and recovery options for the BSRW are limited, despite its being previously considered to have potential for energy production due to favourable gas characteristics (Agar et al. 2018; Eke et al. 2020). BSRW as a feedstock is however too heterogeneous and comprises high ash and moisture content making it unsuitable for a commercial pyrolysis. In this study, we have hypothesized that BSRW after the removal of the less than 10 mm fraction, the moisture and ash content would be reduced and the feasibility of the pyrolysis process would be improved, as well as obtaining a higher quality biochar.

**Fig. 1:**
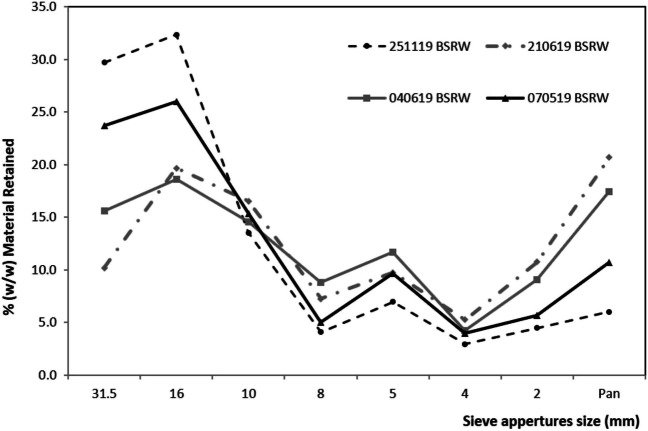
Particle size distribution in BSRW samples (w/w% DM)

**Fig. 2:**
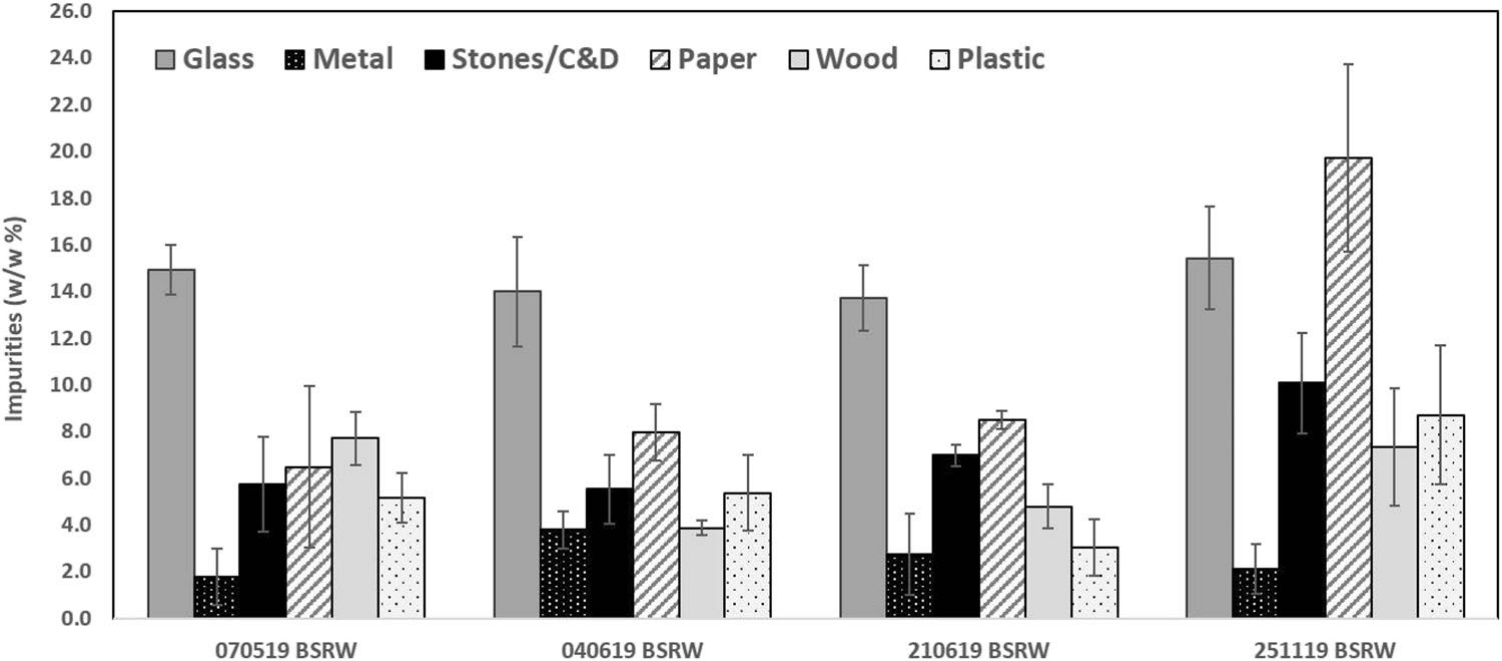
Composition of impurities in the BSRW samples (w/w% DM)

Analysis of the particle size distribution (Fig. 1) showed that the material retained above 10 mm ranged between 50 and 76%, representing at least half of the material currently being landfilled. It is noted that the particle size is quite variable probably due to compositing mechanical operations and/or source of the waste. Nevertheless, it has the potential to recover at least 50% of residual organic waste. Non-organic constituents in the municipal residual waste are rarely studied due to the sluggish sorting and identification process and they are currently included as BSRW. Knowledge of the quantity and type of inert content is important to gain a deeper understanding of the combustible and inert fractions and to assess the post-treatment processes potentially needed. The impurities content in the feedstock (Fig. 2) corresponds to almost 50% of the 10–40 mm BSRW material, greatly limiting the reuse options of this waste. Inert content (e.g. glass, stones and metal) ranges 22–24% of the BSRW and presents lower variability between samples. Inerts are the major unwanted constituents found in the BSRW, corroborating several prior studies (Montejo et al. 2010; Sharifi and Renella 2015). The removal of the inert content from the biochar, although necessary, is considered relatively simple due to the density difference between both materials. The combustible material, such as plastic, paper and wood content of the BSRW 10–40 mm was more variable and ranged from 3 to 9%, 7 to 20% and 4 to 8%, respectively. The content of these materials in the pyrolysis feedstock is desirable, particularly plastics, since they improve its calorific value (Eke et al. 2020). Although high contents of wood and paper can contribute to absorbing moisture and affect the efficiency of gas production in the pyrolysis process (Chen et al. 2005).

The higher heating value (HHV) of BSRW ranged between 13.1 and 15.6 MJ/kg^1^ (Table 3). Ash and moisture content observed were on average 26 ± 7% and 14 ± 2%, respectively. HHV was similar to values reported in the literature (Agar et al. 2018; Eke et al. 2020; Yang et al. 2018) for mixed waste, whereas moisture and ash were lower than reported (Kwapinska et al. 2020; Yang et al. 2018).

**Table 3:** Calorific value, proximate and elemental characterization of BSRW 10–40-mm samples

Parameters % (w/w) as received basis	BSRW 10–40 mm						Compost Oversize
	070519	040619	210619	291019	251119	021219	
Ash	18.9	27.6	19.0	22.4	31.4	36.9	5.6
Moisture	14.8	9.9	15.4	16.7	15.3	12.4	46.7
Volatile matter	57.9	56.7	52.6	52.3	47.7	39.6	37.6
HHV (MJ/kg)	15.4	15.6	15.0	14.5	13.1	13.2	9.9
Sulphur	0.2	0.2	0.3	0.3	0.4	0.4	< 0.1
Chlorine	0.9	0.8	0.8	0.5	0.4	0.3	0.2
Carbon	36.4	40.5	36.6	37.0	35.9	32.4	24.7
Hydrogen	4.5	5	4.4	4.7	4.2	3.3	2.8
Nitrogen	1.2	1.5	1.3	1.7	1.8	1.7	0.5
Oxygen	23.0	14.4	22.2	16.7	10.6	12.5	18.4
O/C molar ratio	0.5	0.3	0.4	0.3	0.2	0.3	0.5
H/C molar ratio	1.5	1.5	1.4	1.6	1.4	1.2	1.3

Analysis of two composite BSRW 0–10-mm samples showed that ash and moisture decreased 1.5-fold and 1.3-fold compared to BSRW without fraction separation, respectively (data not shown), confirming that the removal of the 10 mm fraction improved the properties of the pyrolysis feedstock. Analysis of variance applied to summer and winter samples showed that the HHV was 1 to 2 MJ/kg higher (*F* = 12.81, *p* = 0.023) and chlorine content almost doubled (*F* = 53.34, *p* = 0.0012) in the summer samples whereas the nitrogen content increased 20–28 wt.% (*F* = 14.64, *p* = 0.019) in winter samples (Table 3). Although the interpretation of seasonal analysis needs to be taken with care, due to the small number of samples and known heterogeneity of the feedstock, it shows that the changes in the organic household waste within seasons could affect the pyrolysis process. The season variability in relation to calorific value could be linked with the composition of the waste and changes in the type of food being disposed (Hanc et al. 2011; Maroušek et al. 2020). Winter samples presented the less favourable characteristics for pyrolysis, with higher ash and moisture content. Due to these changes, the samples collected in the winter were chosen to perform the pyrolysis study.

### Pyrolysis products yield

**Fig. 3:**
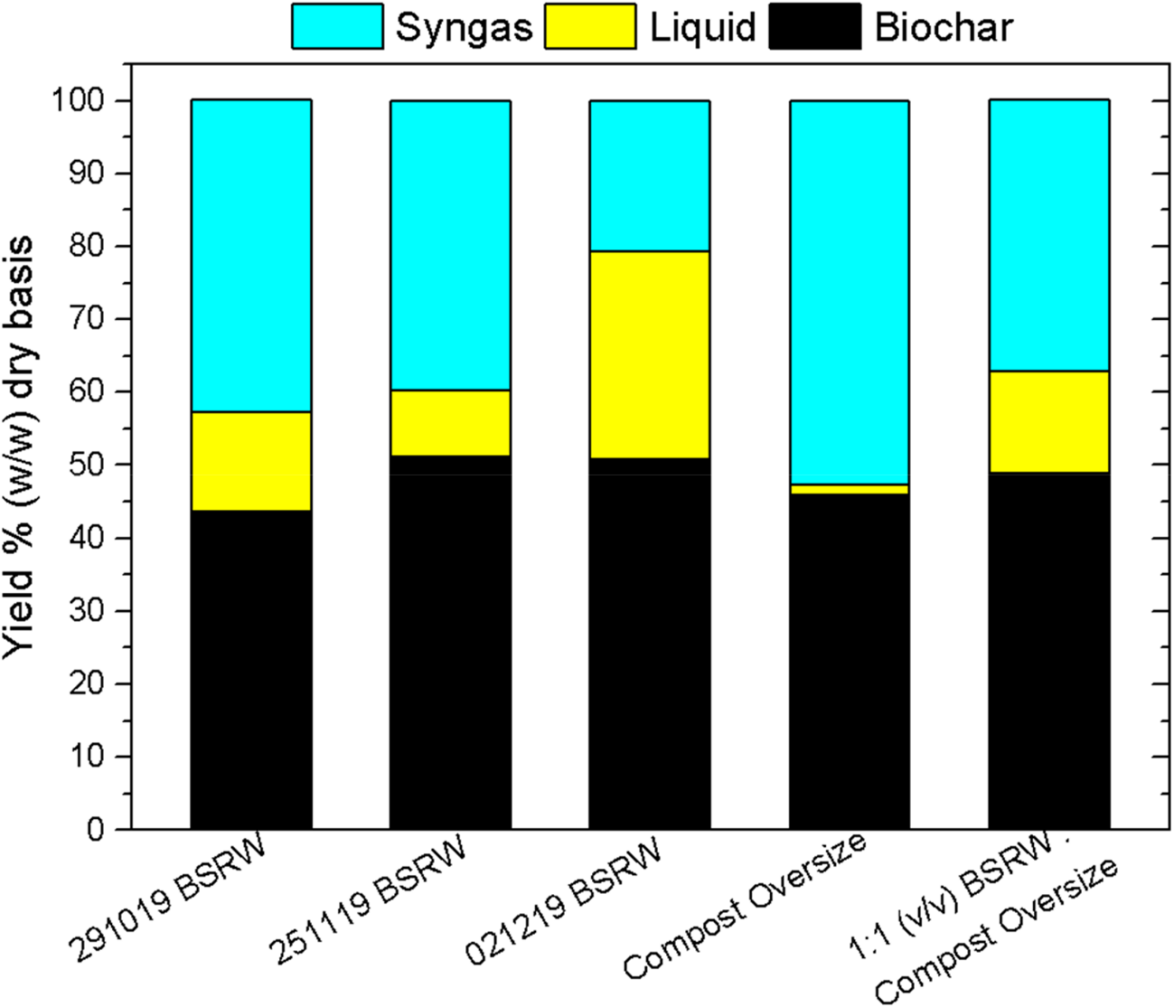
Product distribution on a dry basis from pyrolysis of BSRW samples, Compost Oversize and BSRW to Compost Oversize blend, at 700 °C for 10 min

The three BSRW 10–40 mm collected in winter were chosen for this pyrolysis experiments, since they represented the less favoured characterization. Alongside, a sample of Compost Oversize (> 20 mm) and a sample of 1:1 BSRW 10–40 mm and Compost Oversized (v/v) were also pyrolyzed. The yield of the pyrolysis products presented by the pyrolysis of BSRW 10–40 mm was quite variable, in particular syngas and liquid generated (on as measured basis in Table 4 and on a dry basis in Fig. 3). The char yield, across all samples is relatively even, ranging from even 44 to 51%, whereas gas yield ranged from 21 to 53% on a dry basis. Char yield is consistent with the ash measured in the feedstock prior to pyrolysis (Table 4). Liquid and gas yields presented high variability between the samples. These are mainly reliant on the variable moisture content of the feedstock (Kwapinska et al. 2020) and volatile matter content (Table 3). In this study, the tested feedstock BSRW was pre-treated prior to pyrolysis (inert removal, size reduction, storage frozen) altering its moisture and ash content (Table 4). Despite this limitation, the aim of the study was to generate a feedstock with moisture content < 30%, to make the process technically applicable for the BSRW 10–40 mm. An increased gas fraction is expected in a scaled-up pyrolysis setup, since the vapours in the lab scale equipment condensate immediately after release, generating a liquid fraction. A longer residence time allows for cracking of higher molecular weight molecules, improving the gas yield of the process (Kwapinska et al. 2020). Analysis concluded that the BSRW 10–40 mm is suitable as a pyrolysis feedstock and a pilot scale pyrolysis could be carried out, where the feedstock would be homogenized by reducing its size and inert content should be remove after the process.

**Table 4:** Moisture and ash content of samples measured prior pyrolysis (after defrosting). Yields of pyrolysis products as measured obtained at a temperature of 700 °C and residence time of 10 min

	Samples				
	291019	251119	021219	Compost Oversize	1:1 BSRW/Compost Oversized
Moisture*, % (w/w)	23.4	26.7	15.1	51.7	40.0
Ash*, % (w/w)	21.2	20.8	27.0	8.0	13.5
Ash*, % (w/w) dry basis	27.7	28.4	31.8	16.6	22.1
Biochar yield, % (w/w)	33.4 ± 2.3	51.2 ± 3.6	50.7 ± 4.5	22.2 ± 1.9	27.2 ± 5.4
Liquid yield, % (w/w)	33.8	9.1	28.6	52.4	47.6
Syngas yield, % (w/w)	32.8	39.7	20.7	25.5	25.1

^*^Measured on the day of pyrolysis after feedstock samples defrosting

### Pyrolysis syngas

**Fig. 4:**
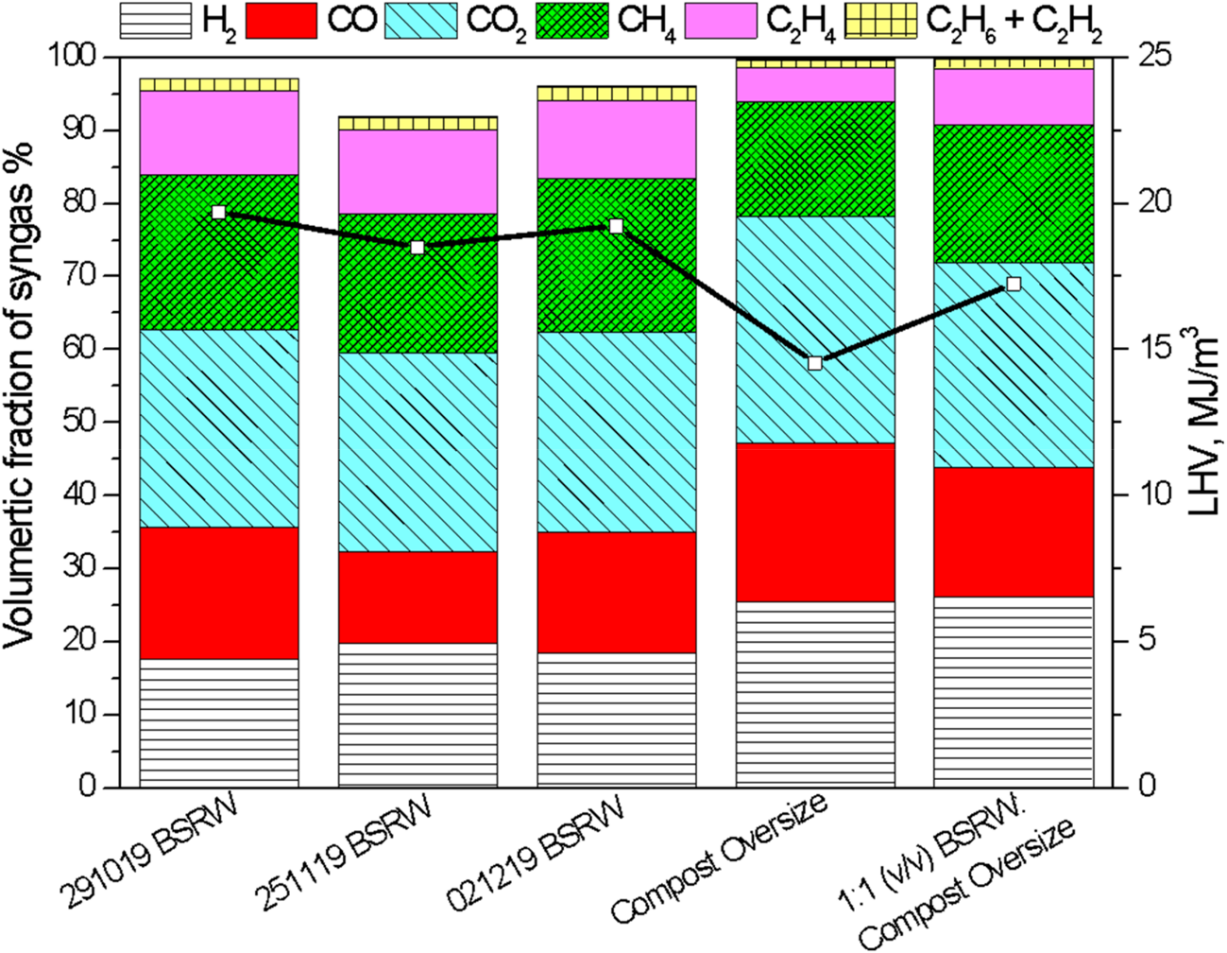
Syngas composition on N_2_ and O_2_ free basis and heating value

Four compounds, carbon dioxide (CO_2_), hydrogen (H_2_), methane (CH_4_) and carbon monoxide (CO), comprised 75% of the syngas produced from the pyrolysis of BSRW. Other gas compounds are released during pyrolysis process such as ammonia (NH_3_), hydrogen cyanide (HCN) and hydrogen sulphide (H_2_S) but those were not measured in this study but certainly are present in the gas. Although syngas composition across the samples analyzed was similar regardless of the use of Compost Oversize (Fig. 4), the latter samples showed lower calorific value. BSRW samples generated syngas with a heating value of 18 MJ/m^3^. Pyrolysis temperature, feedstock moisture content or pre-treatment can affect the composition and heating value of the syngas (Agar et al. 2018; Eke et al. 2020; Palma et al. 2020; Yang et al. 2018). BSRW after composting and removal of the < 10 mm fraction contributed to an improved calorific value and its potential as pyrolysis feedstock.

#### Mass balance of sulphur and nitrogen

The release of sulphur and nitrogen-containing compounds during pyrolysis of residual organic waste is important for further use of syngas/hot pyrolysis vapours (gases and condensable compounds) (Czajczyńska et al. 2017). In this study, the content of H_2_S, NH_3_ and HCN in syngas was not measured but the total amount of sulphur and nitrogen released from BSRW feedstock was estimated from the mass balance calculations based on experimental results (Fig. 3, Tables 3 and 7) and is presented in Table 5.

**Table 5:** Amount of nitrogen and sulphur that remained in biochar or was release into the gas phase during pyrolysis at 700 °C and residence time 10 min

Samples	% of nitrogen remaining in biochar	g N/kg of samples released as gas, dry basis	% of sulphur remaining in biochar	mg S/kg of samples released as gas, dry basis
291019	21.3	15.7	102.5	-
251119	15.0	18.5	119.5	-
021219	24.7	14.4	94.3	285.8
Compost Oversize	40.5	6.0	70.4	444.3

An initial sulphur content of 0.34–0.5% (on a dry basis) was detected in the BSRW samples, of which 94% remained in the biochar and was concentrated. Only about 6% of sulphur was released in a gaseous form most likely as H_2_S. The amount of sulphur remaining in the biochar was much higher compared to a previous study (Kwapinska et al. 2020).

An initial nitrogen content of 1.9–2.2% (on a dry basis) was observed in the BSRW samples, of which 15–24% remained in the biochar, accordingly the remaining 76–85% was released as gases or as a condensable (e.g. tar) or water-soluble compounds. These results are consistent with previous observations (Kwapinska et al. 2020). The Compost Oversize compare to BSRW samples contained less than half of sulphur (0.15% on a dry basis) and nitrogen (1% on a dry basis) of which 70% and 60%, respectively, were released as NH_3_, HCN and H_2_S.

Pyrolysis gas needs to be treated before utilization in combustion unit to avoid NO_x_ emissions (Czajczyńska et al. 2022).

### Energy balance

A simplified energy balance was performed to assess energy potential in hot pyrolysis gas. A scenario in which biochar is used as a conditioner for land application while the hot syngas is combusted to provide the heat required for pyrolysis of BSRW was evaluated. The potential energy obtained from the combustion of the hot syngas (hot pyrolysis vapours) was determined as the difference between the energy input with BSRW stream and the energy output of biochar stream. The energy input with the feedstock was determined by multiplying the LHV of BSRW (calculated based on information provided in Table 3) and the mass of 10 kg of BSRW as received. The energy output sustained with biochar was calculated from the LHV and biochar yield. The LHV of biochars were calculated from the elemental composition presented in Table 6 (Buckley 1991). The results of calculations are provided in Table 6.

**Table 6:** Potential combustion energy in pyrolysis products (assumption: processing of 10 kg of BSRW at the same conditions as batch laboratory scale reactor)

Samples	Input stream	Output streams	
	MJ (%)	Biochar, MJ (%)	Hot pyrolysis vapours (non-condensable gases and condensable organic and water-soluble fraction) (by difference), MJ (%)
BSRW 291019	118 (100%)	40 (33.5%)	78 (66.5%)
BSRW 251119	99 (100%)	40 (40.0%)	59 (60.0%)
BSRW 021219	117 (100%)	55 (47.2%)	61 (52.8%)

The total energy entering the pyrolysis system with the BSRW samples varied since it is a function of moisture content that on the day of pyrolysis tests was from 15 to 26%.

The potential thermal energy released from the combustion of the hot syngas was from 53 to 67% of the energy input with BSRW feedstock.

Thus, most of the initial energy content of the BSRW samples was converted into the hot pyrolysis gas for samples collected in October and November. However, for the sample from December, the amount of energy preserved in the hot gas was the lowest. This finding may reflect seasonal variability in OFMSW composition and/or the effect of weather conditions on compositing. The results on energy balance should be viewed as indicative. It was recently reported that due to the longer residence time and a better heat transfer the yield of biochar at pilot scale operation was lower and the gas yield higher compared to the laboratory tests (Kwapinska et al. 2021). Therefore, in this study, the amount of energy preserved in the hot syngas is considered as a minimum, while the energy in the biochar as a maximum. In these calculations, combustion efficiency and heat loses were not accounted for.  However, there is a strong indication that pyrolysis of BSRW may be energetically self-sustaining even for less favourable samples collected in autumn. This is in line with a threshold value of 10 MJ/kg proposed for MSW feedstocks to ensure energy self-sufficient pyrolysis (Lu et al. 2020a).

### Characterization and end-uses of the biochar

This study tested the biochar from pyrolysis of BSRW (10–40 mm) for two main applications, as an absorbent, using BET surface area, and in land use. We hypothesized that the removal of the less than 10 mm fraction of the BSRW would decrease the heavy metal concentration, since they are accumulated in the small size particles. To evaluate the biochar for agriculture or land use, parameters such as pH, electrical conductivity, organic matter and macronutrients (N, P, K, Mg and Ca) content (Table 7) were used. Biochar pH is alkaline due to the formation of carbonates upon pyrolysis (Tomczyk et al. 2020). This alkaline profile and the content of cations (Ca, K, Mg) could contribute to the use of biochar as a liming agent (Fawzy et al. 2022). Biochar has been shown to contribute to soil carbon build up, due to the high and stable carbon content (Callegari and Capodaglio 2018; EBC 2020). In this study biochar from BSRW samples presented a carbon content ranging between 30 and 34%. When BSRW was pyrolyzed with Compost Oversize, the carbon content of the biochar increased to > 50%. The molar hydrogen to carbon ratio for all biochars was lower than 0.7 (Table 7) indicating high carbon sequestration potential (Enders et al. 2012). The BET surface area of the biochar generated ranged between 17.3 and 32.8 m^2^/g, showing a similar surface area to prior studies (Jin et al. 2014) and MSW (Ndirangu et al. 2019) with potential for wastewater treatment. Prior studies show that biochar produced from OFMSW is suitable to remove organic pollutants from landfill leachate (Jayawardhana et al. 2019; Jin et al. 2014). Complete insights on the adsorption performance of biochar generated from BSRW are needed to understand the efficiency of biochar in the removal of pollutants from wastewater.

**Table 7:** Physical and nutritional properties of the biochar produced in the pyrolysis experiments at 700 °C for 10 min

Biochar properties, % (as analyzed)	BSRW, 291019	BSRW, 251119	BSRW, 021219	Compost Oversize	1:1 BSRW/Compost Oversize
pH	10.6	10.6	10.2	9.80	10.2
Electrical conductivity (µs/cm)	1845	1427	1979	527	1979
Organic matter	43.9	39.2	25.8	67.7	50.4
Moisture	2.5	2.3	2.1	2.1	2.3
Ash	55.6	62.3	52.8	37.2	58.4
Sulphur	0.8	1.0	2.0	0.2	0.4
Carbon	29.9	30.6	34.1	58.0	51.1
Hydrogen	0.83	0.6	0.6	1.9	1.1
Nitrogen	1.0	0.6	0.9	0.9	1.0
Phosphorus	0.8	0.70	0.6	0.19	0.5
Potassium	1.5	1.8	1.7	1.86	1.8
Magnesium	0.9	1.1	1.0	0.20	0.4
Calcium	9.1	14.7	8.8	3.0	6.1
BET surface area m^2^/g	17.9	26.1	32.8	17.3	27.4
H/C molar ratio	0.3	0.2	0.2	0.4	0.3
HHV (MJ/kg),* dry basis	12.0	10.7	13.0	22.4	18.2
LHV (MJ/kg), dry basis	11.8	10.5	12.8	22.2	17.9

^*^Calculated HHV_Milne_ = 0.341·C + 1.322·H − 0.12·O − 0.12·N + 0.0686·S − 0.0153·ash

The heavy metal concentrations in the BSRW biochar complied with the allowable European concentrations produced from source-segregated wastes (Table 8). Biochar produced from BSRW is likely more suitable to be used in non-land applications. Copper is very variable in the biochar samples and a wider study would need to be carried out. Biochar obtained from the pyrolysis of Compost Oversize, a source-segregated stream, and the BSRW blended with Compost Oversize has the potential to be used in land application without the risk of heavy metal contamination. Organic pollutants were not evaluated in the produced biochar samples; however, the organic pollutants content in OFMSW is relatively low and subject to degradation during the composting process (Graça et al. 2021). The pyrolysis process allows the production of a clean organic amendment and the transformation of undesirable materials, such as plastics. The inert content of the biochar (glass, metal, stones) is easily removed using the difference of densities between the materials. Despite these promising results, the authors acknowledge that the characterization of the biochar requires a larger number of samples, due to the high variability between some of the evaluated parameters.

**Table 8:** Heavy metal (mg/kg DM) content in the biochar produced in the pyrolysis experiments at 700 °C for 10 min

		Arsenic	Cadmium		Chromium	Copper	Lead	Mercury	Nickel	Zinc
BSRW 10–40 mm	291019	3.8	1.3		87.9	175	53	< 0.1	27.2	447
	251119	5.5	1.3		30.5	341	163	< 0.1	45.7	492
	021219	8.4	1.5		34.1	464	61	< 0.1	69.9	470
	Av. (STD)	5.9 ± 1.9	1.4 ± 0.1		51.8 ± 2 6.3	327 ± 118	92 ± 51	< 0.1	47.6 ± 17.5	470 ± 18.4
Compost Oversize		2.8	0.5		5.1	27.5	17	< 0.1	7.0	100
1:1 BSRW/Compost Oversize		9.3	8.5		68.7	186	49	< 0.1	38.4	368
Biochar standards										
EBC, 2012	Agrobio	13		0.7	70	70	45	0.4	25	200
	Agro	13		1.5	90	100	150	1	50	400
	Material	15		5	250	250	250	1	250	750

## Limitations of pyrolysis technology for BSRW

Biostabilised residual waste (BSRW) is currently used as landfill cover. However, BSRW is set to lose its “recycled” status under the revised EU waste framework, with probably tighter restrictions and increased costs placed for the landfilling of organic waste. Therefore, there is a need to look for alternative ways of BSRW management. Generally, as BSRW originates from MSW (due to inappropriate source segregation), it contains a lot of impurities such as glass, metal, stones, wood, paper and plastic (Agar et al. 2018). The 10–40 mm BSRW fraction contains up to 50% of impurities (“Feedstock properties” section) besides the organic components. On the one hand, inerts (glass, metal and stones) may pose a challenge for a potential pyrolysis feeding system; on the other hand, they could be easier to separate them from biochar due to difference in density.

Our results indicate that BSRW can be used for pyrolysis when separated into two main fractions. Although pyrolysis of the 10–40 mm BSRW fraction may be energetically self-sustaining as it transforms the organic waste mixed with plastics and paper into combustible vapours/syngas (“Energy balance” section) potential applications of biochar are rather uncertain as they will greatly depend on the presence of residual impurities, e.g. very small pieces of glass.

The number of samples tested and the laboratory-scale pyrolysis that only allows for a limited size of pyrolyzed material can confound the proportions of biochar and vapours/syngas, thus affecting the overall mass and energy balances (“Pyrolysis products yield” and “Energy balance” sections). To produce a more robust scenario for the waste management industry application of pyrolysis of BSRW, a transposition of the current research to a pilot-scale pyrolysis would allow for inferences about financial feasibility.

## Conclusions

Organic waste is still largely disposed of in the general waste, making up for a large portion of the municipal solid waste. This organic waste, once mixed with other wastes, is unsuitable for high-end use and currently being composted and used as daily cover in landfills. As biostabilised residual waste (BSRW) is a very heterogenous material with very high ash content but low calorific value the separation of BSRW into two fractions and the removal of the < 10 mm fraction produced material appropriate for pyrolysis. Seasonal variability in BSRW properties was observed, winter samples are characterized by lower calorific value. In this study, we pyrolyzed the 10–40 mm fraction of BSRW (winter sample) that on its own would half the amount of BSRW currently being landfilled. Laboratory scale tests indicate an energetically sustainable process when hot pyrolysis vapours are directly combusted. Pyrolysis transforms BSRW into biochar, whose yield is from 30 to 50% of the original feedstock, thus allowing for a significant reduction of the disposal. Heavy metals are concentrated in the biochar. Blending BSRW with Compost Oversize from segregated green waste reduces heavy metal content of produced biochar. Providing, the residual impurities could be effectively separated from biochar produced from a blend of BSRW and Compost Oversize, it has the potential to be used in land applications.

The presented process indicates a promising concept to be applied to the recovery of BSRW; however, the financial feasibility needs to be further accessed by upscaling the pyrolysis.
